# Cell Survival Signalling through PPARδ and Arachidonic Acid Metabolites in Neuroblastoma

**DOI:** 10.1371/journal.pone.0068859

**Published:** 2013-07-09

**Authors:** Emma Bell, Frida Ponthan, Claire Whitworth, Frank Westermann, Huw Thomas, Christopher P. F. Redfern

**Affiliations:** 1 Northern Institute for Cancer Research, Newcastle University, Newcastle upon Tyne, United Kingdom; 2 Division of Tumor Genetics, German Cancer Research Center (DKFZ), Heidelberg, Germany; University of Hawaii Cancer Center, United States of America

## Abstract

Retinoic acid (RA) has paradoxical effects on cancer cells: promoting cell death, differentiation and cell cycle arrest, or cell survival and proliferation. Arachidonic acid (AA) release occurs in response to RA treatment and, therefore, AA and its downstream metabolites may be involved in cell survival signalling. To test this, we inhibited phospholipase A2-mediated AA release, cyclooxygenases and lipoxygenases with small-molecule inhibitors to determine if this would sensitise cells to cell death after RA treatment. The data suggest that, in response to RA, phospholipase A2-mediated release of AA and subsequent metabolism by lipoxygenases is important for cell survival. Evidence from gene expression reporter assays and PPARδ knockdown suggests that lipoxygenase metabolites activate PPARδ. The involvement of PPARδ in cell survival is supported by results of experiments with the PPARδ inhibitor GSK0660 and siRNA-mediated knockdown. Quantitative reverse transcriptase PCR studies demonstrated that inhibition of 5-lipoxygenase after RA treatment resulted in a strong up-regulation of mRNA for PPARδ2, a putative inhibitory PPARδ isoform. Over-expression of PPARδ2 using a tetracycline-inducible system in neuroblastoma cells reduced proliferation and induced cell death. These data provide evidence linking lipoxygenases and PPARδ in a cell survival-signalling mechanism and suggest new drug-development targets for malignant and hyper-proliferative diseases.

## Introduction

Retinoic acid (RA) is a biologically-active vitamin A metabolite used in the treatment of neuroblastoma and acute promyelocytic leukaemia [1]. *In vitro,* RA induces growth arrest, down-regulation of MYCN expression [2] and differentiation in neuroblastoma cells [3]. Paradoxically, RA can promote increased proliferation and cell survival in certain cell types [4], [5]. Like other anticancer agents such as cisplatin and tamoxifen, RA induces arachidonic acid (AA) release in cancer cells [6]–[9], and this may promote cell survival under conditions of cell stress. Furthermore, celecoxib, a non-steroidal anti-inflammatory drug and cyclooxygenase (COX2) inhibitor which inhibits the metabolism of AA, potentiates the effects of both RA and cytotoxic drugs in neuroblastoma cells [10]–[12].

RA has been reported to activate Peroxisome Proliferator-Activated Receptor (PPAR) δ, a ligand-activated transcription factor controlling cell growth and proliferation and important for cell survival [13]. RA is thought to be transported into the nucleus by cellular retinoic acid binding proteins (CRABP) or fatty acid binding protein 5 (FABP5) and it has been proposed that CRABP2 mediates RA transfer to RA receptors (RAR) to promote differentiation or apoptosis, whereas FABP5 mediates RA transfer to PPARδ heterodimers promoting cell survival [14]. Evidence for the direct activation of PPARδ by RA is controversial, with later studies suggesting that RA does not directly bind to PPARδ or activate PPAR target genes [15]–[17]. Nevertheless, there may well be interactions between RAR and PPARδ signalling pathways in development; for example, it has recently been suggested that neural differentiation is regulated by an RAR-mediated commitment phase followed by the promotion of differentiation via a PPARδ-mediated up-regulation of PDK1 [18]. The role of PPARδ in cell signalling is likely to be complex; five different mRNA isoforms of PPARδ have been described, with PPARδ1 and PPARδ2 being the most abundantly expressed in human tissues; although PPARδ2 has been suggested to represent an inhibitory isoform, a translational product has yet to be identified [18].

Given the activity of celecoxib in inducing cell death in combination with RA, it is possible that AA metabolites are important in promoting cell survival and may interact with RAR- and/or PPARδ-mediated signalling. To test this hypothesis and elucidate the mechanism of interaction between RA and celecoxib, we investigated the effect of inhibiting AA release, cyclooxygenases and lipooxygenases on the survival of neuroblastoma cells after RA treatment. The data suggest that 5-lipoxygenase (5-LO) inhibition sensitises neuroblastoma cells to apoptosis and that celecoxib promotes RA-induced neuroblastoma cell death *in vitro* through the inhibition of 5-LO. Further experiments to clarify the potential role of 5-LO suggest that the 5-LO product 5-oxo-eicosatetraenoic acid (5-oxo-ETE) mediates cell survival through PPARδ.

## Materials and Methods

### Established Cell Lines and Culture Conditions

SH-SY5Y [19], NGP [20] and NB69 [21] neuroblastoma cells were grown in 1∶1 DMEM/F12 (Sigma-Aldrich, Poole, UK) supplemented with 10% FBS (Invitrogen, Paisley, UK) at 37°C in 5% CO_2_. SH-SY5Y^tet12^ cells [22] were grown in DMEM/F12 1:1 10% FBS, supplemented with blasticidin (5 µg/ml; Invitrogen).

### Chemicals

All-*trans* RA (ATRA), AACOCF3, GSK0660, MK886 and Prostaglandin E2 (PGE2) were from Sigma-Aldrich, PD-146176 from Enzo Life Sciences (Farmindale, NY), celecoxib from Pfizer (NY), baicalein, 5-oxo-ETE and leukotriene A4 (LTA4) methyl ester were from Cayman Chemicals (Ann Arbor, MI). LTA4 is stable at 4°C for 24 h; therefore, we used LTA4 methyl ester and performed hydrolysis according to manufacturer’s instructions immediately prior to experiments to convert the stable LTA4 methyl ester into LTA4. L165-041 was from Tocris Bioscience (Ellisville, MO).

### Flow Cytometry

Adherent and non-adherent cells were fixed in 70% ethanol, 30% PBS, then spun down and resuspended in PBS containing 40 µg/ml propidium iodide and 100 µg/ml RNAse A. Fluorescence from excitation at 488 nm with a 15-mW argon laser was monitored at 585±21 nm using a FACScan flow cytometer with CellQuest software (Becton Dickinson, San Jose, CA). Events were counted using a doublet-discriminator parameter to exclude aggregates and a threshold of forward and side scatter was used to exclude debris. Cell-cycle and cell-death analysis was carried out on the same samples using WinMDI (version 2.8, TSRI, La Jolla, CA).

### PPARδ siRNA

10 nM PPARδ SMART-pool siRNA (Dharmacon, Lafayette, CO) was complexed with lipofectamine in OptiMEM (Invitrogen), according to the manufacturer’s instructions. Control siRNAs were a universal scrambled negative control (SCR) (Qiagen, Ontario, Canada). Cells were transfected with siRNA for 24 h prior to treatment with ATRA. Levels of apoptosis were higher in SH-SY5Y cells treated with siRNA (SCR or PPARδ), therefore, for assessing the effects of knockdown on ATRA-induced apoptosis the data were normalised to the zero-ATRA control for each siRNA.

### PPARδ Reporter Assay

Cells were transfected with 667 ng of PPAR response element (PPRE) construct, negative or positive control plasmids from the PPRE cignal dual luciferase reporter assay kit (SAbiosciences, Frederick, MD) using lipofectamine. 24 h after transfection, cells were treated with drug combinations for 2 h. Cells were harvested and transferred to a 96-well plate; 50 µl of Stop and Glow solution (Promega, Madison, WI) was added to each well and luminescence measured on a LB96V Microplate Luminometer (EG & G Berthold, Bad Wildbad, Germany).

### Establishment of a Stable Inducible PPARδ2 Over-expression System in SH-SY5Y Cells

Human PPARδ, mRNA transcript variant 2 cDNA in pOTB7 (IOH5357, Invitrogen) was subcloned into BamH1 and Xho1 sites of pcDNA4/TO (Invitrogen). The construct was transfected into SH-SY5Y^tet12^ cells expressing the TET repressor [22]. After selection using zeocin (250 µg/ml) and blasticidin (5 µg/ml), a mixed population of cells was used for experiments (SH-SY5Y^tet12^PPARδ2). Addition of 1 µg/ml doxycycline to the medium induced PPARδ2 expression (referred to as PPARδ2+ve). Cells grown without doxycycline are referred to as PPARδ2-ve. To confirm results, transient transfections were carried out using the pcDNA4/TO PPARδ2 construct and native SH-SY5Y cells.

### RNA Extraction and Quantitative Reverse Transcriptase PCR for PPARδ

RNA was extracted using the RNeasy mini kit (Qiagen) and DNAse-treated using the DNA-free kit (Qiagen). 1 µg of RNA was reverse transcribed using a high-capacity reverse transcription kit. Levels of PPARδ1 and PPARδ2 were measured using Taqman assays (PPARδ1: Hs00606407_m1 and PPARδ2 Hs02516538_s1) and normalised to endogenous GAPDH (Hs99999905_m1). Quantitative reverse transcriptase **(**qRT-PCR) was carried out using the relative quantification protocol on an ABI Prism 7900HT and analysed using SDS software (Applied Biosystems, Foster City, CA).

### XTT Viability and Cell Growth Assays

The XTT assay (Roche Diagnostics, Basel, Switzerland) was used as previously described [23]. The experimental conditions were based on standard calibration curves to establish the appropriate initial plating density. For viability dose-response curves, cells were seeded at 2 x10^4^ per well into 96-well microtiter plates (Corning, Amsterdam, Netherlands) and left to adhere overnight; inhibitors and ATRA were diluted in OptiMEM to the appropriate concentrations. For growth curves, cells were plated at 5×10^3^ per well, and XTT absorbance was measured on 3 consecutive days. Treated cells were incubated for 24 h before XTT solution was added; plates were read at 480 nm 4 h after XTT addition.

### Western Blotting

Cells were lysed in buffer containing protease inhibitors (Roche Diagnostic, Mannheim, Germany). Protein concentrations were measured using Bradford reagent (Pierce, Rockford, IL) and equal quantities separated by SDS-PAGE, transferred to nylon membranes (Bio-Rad, Hertfordshire, UK) and probed with antibodies against COX2 (Santa Cruz Biotechnology Inc, Santa Cruz, CA), cleaved caspase-3 (Cell Signalling Technologies, Danvers, MA) and β-actin (Sigma-Aldrich). Anti-mouse IgG conjugated with horseradish-peroxidase (Upstate, Billerica, MA, USA) was used as secondary antibody and enhanced chemiluminescence Western blotting detection reagent (GE Healthcare, Buckinghamshire, UK) was used for detection.

### Statistics

Graphs were plotted using Sigmaplot 11.0 (San Jose, CA). Dose-response curves were fitted using the non-linear mixed-effects model fit in the nlme package [24] in R [25]. For other experiments, univariate ANOVA (with contrasts) was performed in SPSS Version 17 (Chicago, IL), and t-tests (unpaired) and linear mixed-effects models (nlme package [24]) were performed using R. Where relevant, combination indices (CI) were calculated using Calcusyn (Biosoft, Cambridge, UK). CI>1 indicates an antagonistic interaction between two drugs and a CI<1 indicates synergy. All errors are expressed as standard error of the mean (SEM). For clinical data, Wilcoxon test was used to assess differential expression in *MYCN-*amplified (all stages) versus non-*MYCN*-amplified (all stages). For Kaplan-Meier survival analysis the best cut off between low and high PPARδ1 expression was determined as the 1st quartile. The log-rank test was used to test whether the two survival curves are different.

## Results

### Inhibition of AA Metabolism Promotes RA-mediated Cell Death

**Figure 1:**
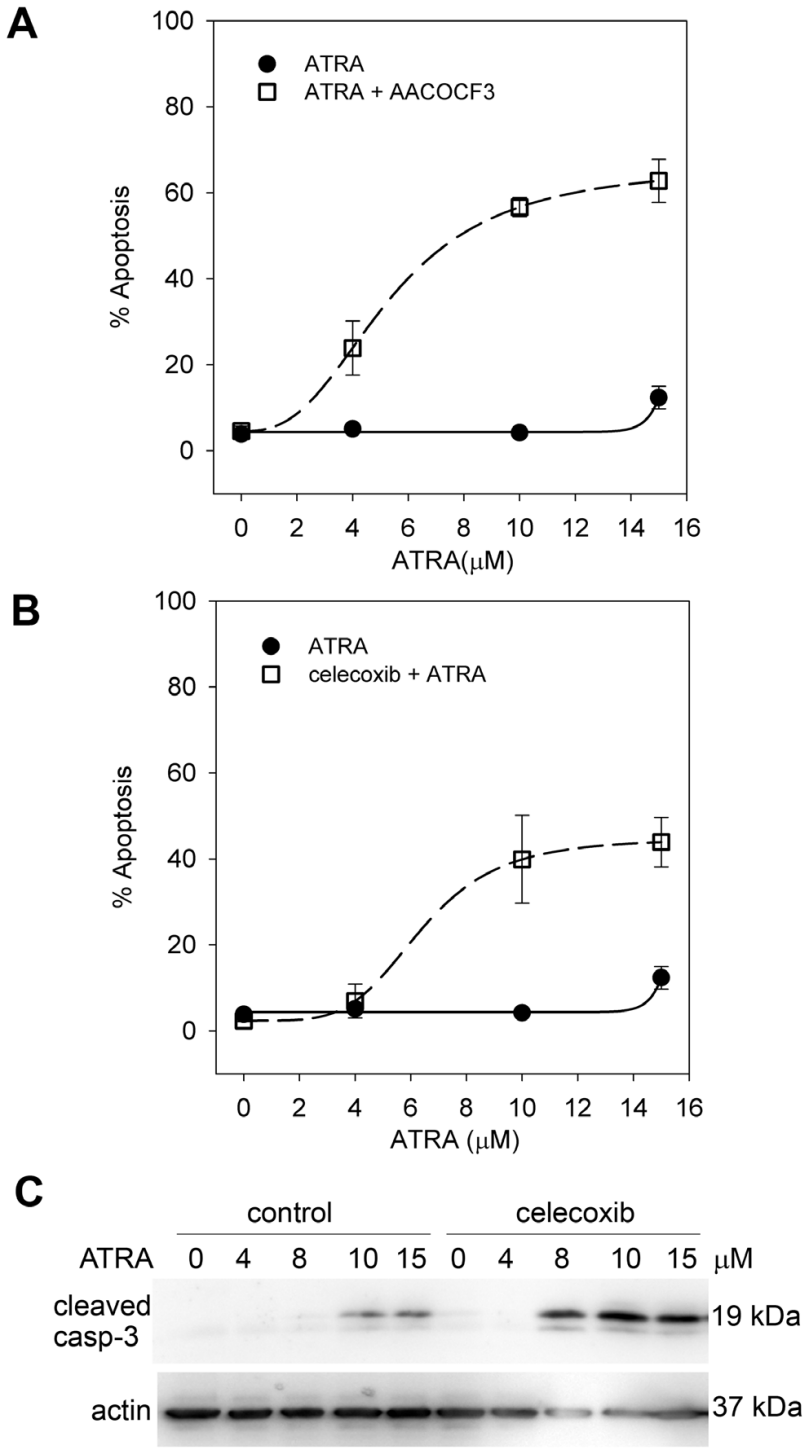
Inhibition of AA metabolism promotes RA mediated cell death. All graphs show data for SH-SY5Y cells. Apoptosis was measured after 24 h of treatment using flow cytometry in (**A**) and (**B**). The apoptotic fraction was determined as the sub-G1 peak. (**A**) Apoptosis of cells treated with ATRA alone (•, solid line) or ATRA in combination with 10 µM of the PLA2 inhibitor AACOCF3 (□, dashed line). Levels of apoptosis were significantly higher in the SHSY5Y cells treated with a combination of ATRA and AACOCF3 (ANOVA, P<0.001). (**B**) Apoptosis of cells treated with ATRA alone (•, solid line) or ATRA in combination with 22 µM celecoxib (□, dashed line). The interaction P-value for ATRA and celecoxib was P = 0.03 (ANOVA). For apoptosis experiments there were three technical replicates for each of three dose response curve experiments. Viability results for the corresponding experiments are summarised in Table 1 with additional viability data for NGP and NB89 cells in Table S1. (**C**) Western blot for cleaved caspase-3, 6 h after treatment with ATRA or ATRA with 22 µM celecoxib, size markers from ladder are shown; the full blot is shown in Figure S3.

Several studies have shown that RA promotes AA release from nuclear membranes [6], [7]. To determine if AA release is important in survival of cells treated with all-*trans* RA (ATRA), we inhibited phospholipase A2 (PLA2) using 10 µM AACOCF3 prior to ATRA treatment (Figure 1A & Table 1); this concentration of AACOCF3 has been used previously to inhibit AA release in neuroblastoma cell lines [26]. Flow-cytometry was used to estimate the proportion of cells within the sub G_1_ peak as a measure of cell death in SH-SY5Y cells; the results were confirmed by XTT viability assays in SH-SY5Y, NGP and NB69 cells. The data showed that AACOCF3 facilitated ATRA-dose-dependent cell death in all three neuroblastoma cell lines (Figure 1A; Table 1 and Table S1). These results suggest that AA is critical for cell survival after ATRA treatment.

**Table 1:** Viability of SH-SY5Y neuroblastoma cells treated with ATRA (dose-response) alone or in the presence of AA-signalling inhibitors.

Drug (concentration)	Target	Drug alone (% viability)	% viability using drug incombination with 15 µMATRA	IC_50_ (µM) ATRA in combinationwith drug
ATRA (0.5 µM to 15 µM)		95.53±2.05*	95.53±2.05*	7.88±0.002
AACOCF3 (10 µM)	PLA_2_	76.88±14.17	0	7.56±1.05
Celecoxib (22 µM)	COX2/5-LO	89.54±6.97	0	4.27±2.03
MK886 (1 µM)	FLAP (5-LO)	59.7±12.7	0	4.9±1.7
GSK0660 (0.2 µM)	PPARδ	88.0±10.6	0	10.2±0.3

* ATRA alone: Viability at 15 µM.

SH-SY5Y cells were pre-treated for 2 h with inhibitor (or vehicle control) at the concentrations specified (left column) prior to the addition of ATRA and viability was measured after 24 h incubation with ATRA in the presence of inhibitor using XTT assays. The % viability of the cells treated with the specified dose of inhibitor alone (middle right column) is given alongside the IC_50_ of ATRA and inhibitor together (right column). Unless otherwise stated, the IC_50_ was calculated from data where 0% viability resulted from the maximal ATRA concentration. For viability experiments, there were 6 technical replicates for each dose-response curve and each dose-response curve was performed three times. Results are mean %±SEM.

AA is a substrate for cycloxygenases and lipoxygenases and is metabolised to prostaglandins or lipoxins, leukotrienes and hydroxy-eicosatetraenoic acids [27], [28]. Therefore, we used small-molecule inhibitors to target cyclooxygenases and lipoxygenases in combination with ATRA. Firstly, we treated neuroblastoma cells with a sub-lethal dose (22 µM) of celecoxib; in combination with ATRA, celecoxib synergistically decreased cell viability in all three cell lines (combination index [CI] = 0.119 for SH-SY5Y cells with 15 µM ATRA; Table 1 & Table S1). Apoptosis data revealed that celecoxib in combination with ATRA increased apoptosis in a dose-dependent manner (Figure 1B). Elevated levels of apoptosis after 24 h in cells treated with ATRA and celecoxib were confirmed by substantially-increased caspase-3 cleavage relative to ATRA alone after 6 h of treatment (Figure 1C). Although cleaved caspase-3 increased in SH-SY5Y cells after treatment with 10 µM or 15 µM of ATRA for 6 h, at 10 µM ATRA this was not linked to a detectable increase in apoptosis at 24 h. Thus, caspase-3 cleavage occurred to a greater extent and with lower concentrations of ATRA in the combination treatment, and corresponded to significantly higher apoptosis at 24 h.

### 5-LO Promotes Cell Survival After RA Treatment

**Figure 2:**
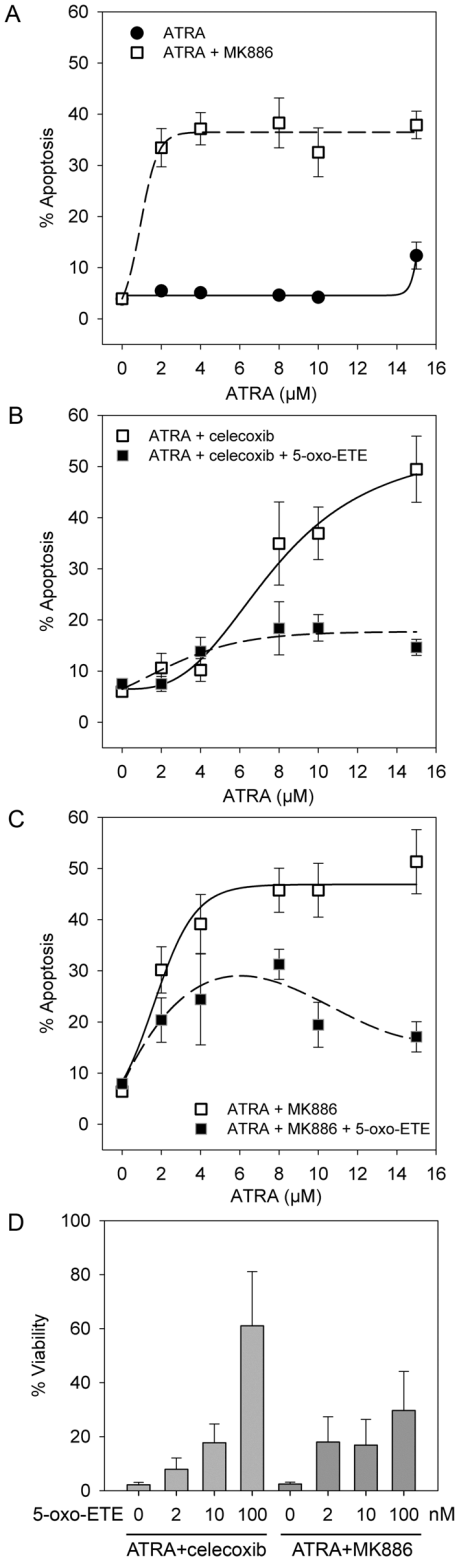
A 5-LO metabolite promotes cell survival after RA treatment. All graphs show data for SH-SY5Y cells. Apoptosis was measured by flow cytometry as the size of the sub-G1 peak. (**A**) Apoptosis of cells treated with increasing doses of ATRA alone (•, solid line) or with 1 µM MK886 (□, dashed line). The interaction P-value for MK886 and ATRA was<0.001 (ANOVA). Data for MK886 alone are in Table 1. (**B**) Apoptosis of cells treated with increasing doses of ATRA in combination with 22 µM celecoxib (□, solid line) or after pre-treatment with 0.1 µM 5-oxo-ETE prior to increasing doses of ATRA in combination with 22 µM celecoxib (▪, dashed line). The interaction between 5-oxo-ETE and celecoxib/ATRA was significant, P = 0.001 (ANOVA). (**C**) Apoptosis of cells treated with increasing doses of ATRA in combination with 1 µM MK886 (□, solid line) or after pre-treatment with 0.1 µM 5-oxo-ETE prior to increasing doses of ATRA in combination with 1 µM MK886 (▪, dashed line). The interaction between 5-oxo-ETE and MK886/ATRA was significant, P = 0.005 (ANOVA). (**D**) Rescue, expressed as % viability, of cells treated with 15 µM ATRA with 22 µM celecoxib (light grey bars) or ATRA with 1 µM MK886 (dark grey bars) by pre-treatment with increasing doses (0, 2 nM, 10 nM or 0.1 µM) 5-oxo-ETE. All error bars are±SEM.

As celecoxib is well known as a COX2 inhibitor, we investigated the role of AA metabolism by COX2 in promoting cell survival after ATRA treatment and concluded that COX2 was not the main cell-survival mediator of ATRA in neuroblastoma cells (Text S1, Figure S1 and Figure S4). Celecoxib also inhibits 5-LO [29] and 5-LO is expressed in neuroblastoma cell lines [26] and tumours [30]. To determine if 5-LO inhibition promotes synergy between celecoxib and ATRA we tested the combination of the 5-LO inhibitor MK886 and ATRA. To become catalytically active, 5-LO binds the 5-LO activating protein FLAP; MK886 also binds FLAP and as a consequence inhibits 5-LO activity [31]. In SH-SY5Y cells, 1 µM MK886 is a sub-lethal dose [22] but apoptosis increased significantly when combined with increasing doses of ATRA (Figure 2A). MK886 in combination with ATRA effectively reduced survival in all three neuroblastoma cell lines (Table 1 and Table S1). We assessed the contribution of AA metabolism by lipoxygenases 12-LO and 15-LO to cell-survival signalling and concluded that 5-LO inhibition was the most effective strategy for promoting cell death in combination with ATRA (Text S2, Table S2).

5-LO oxidises AA to both LTA4 and 5*S*-hydroperoxy- 6,8,11,14-eicosatetraenoic acid; the latter is rapidly reduced and then oxidised by NADP+-dependent 5-hydroxyeicosanoid dehydrogenase (5HEDH) activity to 5-oxo-ETE [32]. If 5-LO metabolites promote cell survival after ATRA treatment, we would predict that pre-incubation with 5-oxo-ETE or LTA4 would rescue cells from ATRA and 5-LO inhibition. In experiments to test this, 5-oxo-ETE partially rescued SH-SY5Y cells from death induced by 22 µM celecoxib and ATRA or 1 µM MK886 and ATRA (Figure 2B and C). Viability assays confirmed the apoptosis results: at the maximum dose of ATRA (15 µM), 5-oxo-ETE increased viability from 5.6%±2.4 to 42.6%±7.06 with the combination of ATRA and celecoxib (Table 2). A similar effect was observed with the combination of 1 µM MK886 and 15 µM ATRA, with viability increasing from 1.3%±1.5 to 22.05%±6.4 in the presence of 5-oxo-ETE (Table 2). The data show that 5-oxo ETE affected levels of cell survival at high concentrations of ATRA (the lower asymptote), and that the effects of 5-oxo-ETE were dose dependent (Figure 2D). In contrast, 0.1 µM LTA4 did not rescue SH-SY5Y cells from celecoxib or MK886 in combination with ATRA (Table 2). We interpret these results to suggest that metabolism of AA by 5-LO promotes cell survival in neuroblastoma cells.

**Table 2:** The effect of AA-signalling agonists and antagonists on the viability of SH-SY5Y neuroblastoma cells treated with ATRA (dose-response) in combination with fixed doses of the 5-LO inhibitors celecoxib or MK886.

Drug combined withATRA dose-response	Agonist/antagonist (0.1 µM)	Viability IC_50_ (µM)	Lower asymptote
Celecoxib 22 µM	none	4.27±2.03	0.26%±0.06
MK886 1 µM	none	4.9±1.7	0.26%±1.6
Celecoxib 22 µM	5-oxo-ETE	7.3±1.4	42.6%±7.06
MK886 1 µM	5-oxo-ETE	4.9±0.9	22.05%±6.4
Celecoxib 22 µM	LTA4	6.9±0.7	8.8%±3.1
MK886 1 µM	LTA4	4.9±0.1	2.1%±0.3
Celecoxib 22 µM	L165-041	12.0±0.5	5.9%±2.4
MK886 1 µM	L165-041	8.4±0.4	1.2%±0.2

SH-SY5Y cells were pre-treated with 0.1 µM 5-oxo-ETE, 0.1 µM LTA4 or 0.1 µM L165-041 2 h prior to treatment with 22 µM celecoxib and an ATRA dose response, or 1 µM MK886 and an ATRA dose response. Viability was measured 24 h after treatment using XTT assays. The IC_50_ values are for the ATRA dose-response with fixed doses of the 5-LO inhibitors celecoxib or MK886 and in the presence of agonist/antagonist or control vehicle, and the lower asymptote reflects the viability of cells at the highest concentrations of ATRA in the dose-response curve. For viability experiments, there were three technical replicates for each of three dose-response experiments. Results are mean %±SEM.

### PPARδ Activation by 5-oxo-ETE is Involved in RA-mediated Cell Survival Signalling

**Figure 3:**
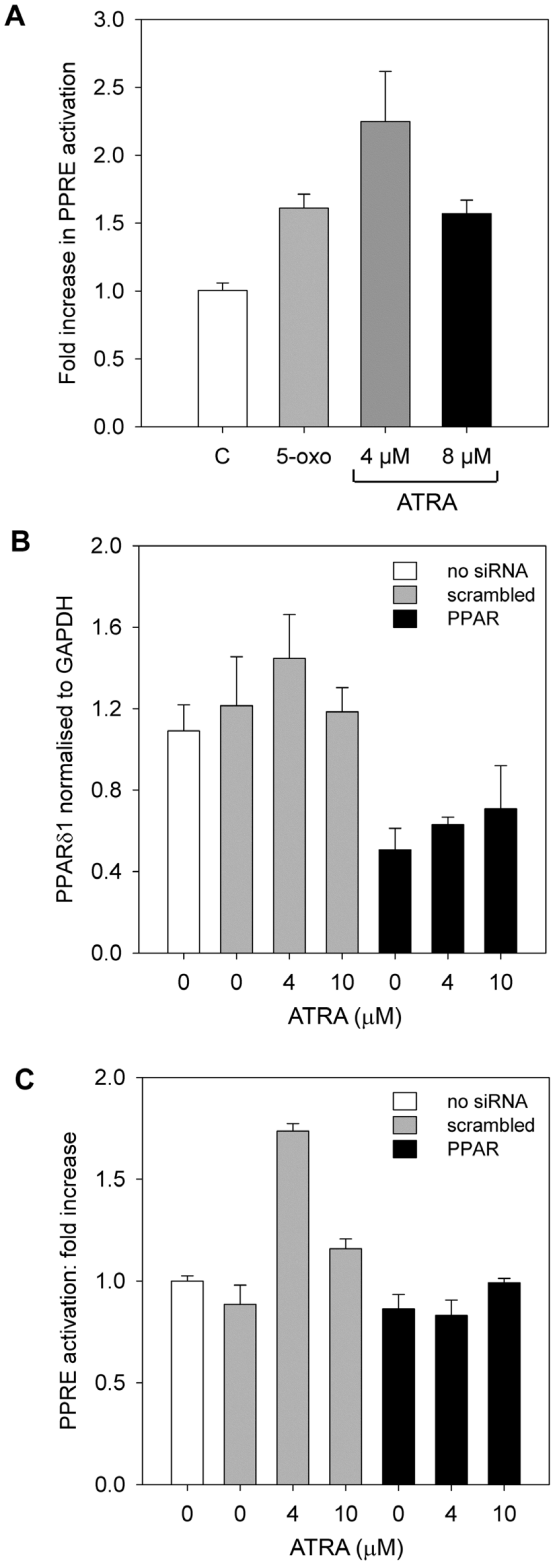
PPRE activation by PPARδ after RA treatment. (**A**) PPRE reporter activation in SH-SY5Y cells 2 h after treatment with control vehicle (white bar), 0.1 µM 5-oxo-ETE (light grey bar), 4 µM ATRA (dark grey bar), or 10 µM ATRA (black bar). PPRE activation was significantly higher with all 3 treatments compared to control (1-way ANOVA with contrasts: P = 0.019, P = 0.01 and P>0.001 for each treatment, respectively). (**B**) PPARδ1 mRNA levels in SH-SY5Y cells transfected with 10 nM PPARδ (black bars) or SCR siRNA (grey bars) for 24 h and then treated with control vehicle (0) or ATRA at 4 µM or 10 µM for 2 h. The SCR control did not significantly affect PPARδ1 expression, compared to the mock-transfected control (white bar; P = 0.566). Across treatments, PPARδ siRNA significantly decreased PPARδ1 mRNA expression compared to SCR and mock-transfected controls (1-way ANOVA, P<0.001). (**C**) PPRE reporter activation in siRNA-transfected SH-SY5Y cells in the presence of ATRA (4 µM or 10 µM) or control vehicle (0). PPARδ (black bars) or SCR (grey bars) siRNA had no effect on the controls (C; 1-way ANOVA with contrasts, P = 0.85). Reporter activation was significantly lower in the PPARδ siRNA-transfected cells after treatment with 4 µM or 10 µM ATRA (1-way ANOVA with contrasts, P<0.001 and P = 0.015, respectively). All error bars are±SEM.

Previous studies have suggested that PPARδ mediates survival response to ATRA [13], [14]. Assays for PPRE binding activity in nuclear extracts from SH-SY5Y cells demonstrated that PPARδ was 4.5- to 6-fold more abundant than PPARα or PPARγ (Figure S2A). PPRE activation increased significantly in SH-SY5Y cells after treatment with 4 or 8 µM ATRA (2.3±0.4 and 1.6±0.01 -fold respectively; Figure 3A). Importantly, 0.1 µM 5-oxo-ETE increased PPRE activation to similar levels observed with 4 and 8 µM ATRA, suggesting that 5-oxo-ETE is substantially more potent than ATRA (Figure 3A). Furthermore, treatment with MK886 reduced PPRE activation in response to ATRA (Figure S2B). Therefore, these results suggest that PPRE activation in response to ATRA may be an indirect effect resulting from stimulation of PPAR by AA metabolites such as 5-oxo-ETE.

PPARδ siRNA knock-down in SH-SY5Y cells was used to determine if PPARδ mediates increased PPRE-binding activity after ATRA treatment. Levels of PPARδ1, the full-length PPARδ transcript, and PPARδ2 were measured 24 h after treatment with siRNA (control samples) and 2 h after subsequent treatment with 4 or 10 µM ATRA (Figure 3B). Before ATRA treatment, PPARδ1 mRNA was reduced by 58%; after treatment with ATRA, PPARδ1 mRNA levels remained between 40–60% compared to the corresponding scrambled (SCR) siRNA control (Figure 3B). The specificity of the commercially-supplied PPARδ siRNA with respect to the PPARδ1 and PPARδ2 isoforms is unknown but there was no significant effect of the PPARδ siRNA on PPARδ2 transcript levels (ANOVA, scrambled versus PPAR siRNA, P = 0.46). There was no significant difference in PPRE reporter-gene activity between the PPARδ and SCR siRNA-treated controls (Figure 3C) but levels of PPRE reporter activity in cells treated with 4 or 10 µM ATRA were significantly lower in the PPARδ siRNA-treated cells (Figure 3C).

### PPARδ Inhibition Promotes Cell Death after ATRA Treatment

**Figure 4:**
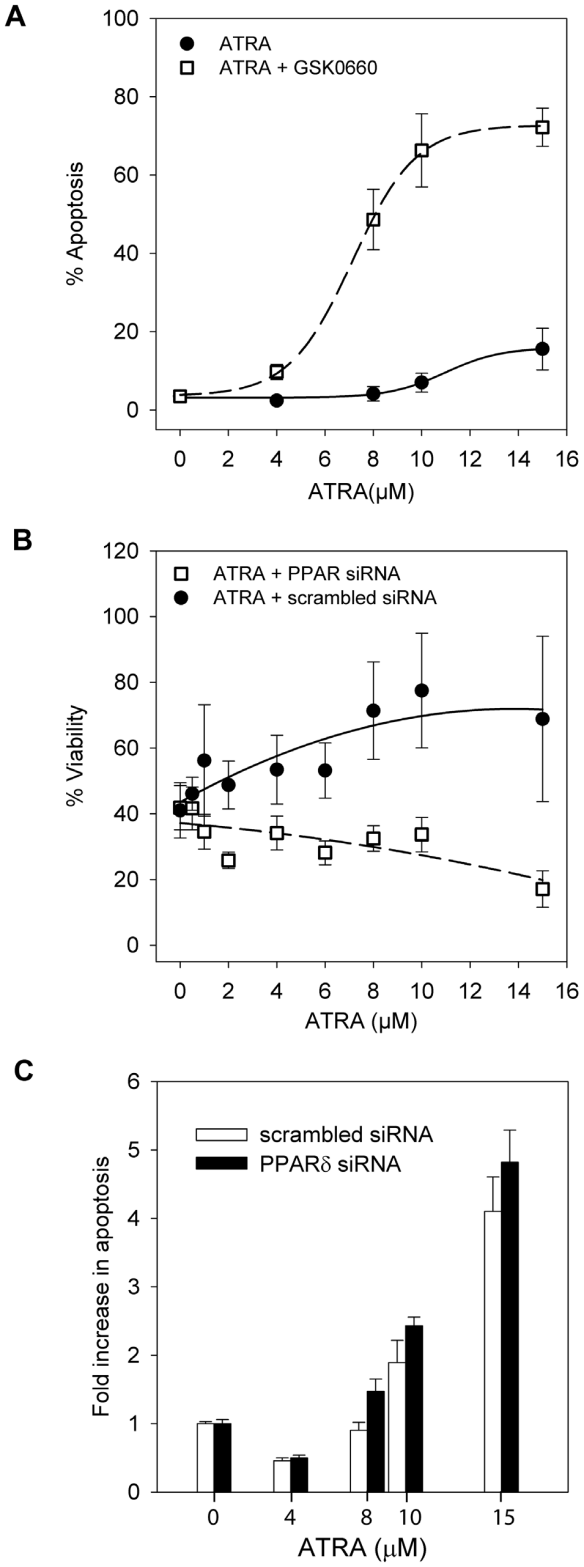
PPARδ inhibition sensitises cells to cell death after treatment with ATRA. All graphs show data for SH-SY5Y cells. (**A**) Apoptosis was measured after 24 h of treatment using flow cytometry. Cells were treated with increasing doses of ATRA (•, solid line) or increasing doses of ATRA in the presence of 0.2 µM GSK0660 (□, dashed line). The interaction P-value for GSK0660 and ATRA was P<0.001(ANOVA). Data for GSK0660 alone are in Table 1. (**B**) Viability determined by XTT assay for cells transfected with PPARδ (□, dashed line) or SCR siRNA (•, solid line) and treated with increasing doses of ATRA. (**C**) Apoptosis of cells transfected with PPARδ (black bars) or SCR siRNA (white bars) and treated with 0, 4 µM, 8 µM or 10 µM ATRA. PPARδ knockdown alone did not increase apoptosis; ATRA at concentrations of 8 µM or above increased apoptosis compared to control (no ATRA) and apoptosis at these concentrations was increased by PPARδ knockdown (Linear mixed effects model, effect of siRNA P = 0.014). The level of apoptosis ranged from a mean of 16% in siRNA-treated cells without ATRA to 67% in cells treated with PPARδ siRNA and 15 µM ATRA. All error bars are±SEM.

To investigate the role of PPARδ in cell survival, PPARδ activity was inhibited prior to ATRA treatment using two different approaches: a small-molecule PPARδ inhibitor, GSK0660, and siRNA-mediated PPARδ knockdown. Apoptosis was significantly increased after cells were treated with a combination of GSK0660 (0.2 µM) and ATRA compared to ATRA alone (Figure 4A). Viability assays confirmed these results with GSK0660 on its own slightly reducing cell viability to 88.0±10.6% of the controls, but substantially decreased viability in combination with increasing doses of ATRA (Table 1). Transfection of SH-SY5Y cells with PPARδ or SCR siRNA decreased the overall viability of the cells, and this was reflected in the reduced viability of the controls (Figure 4B). However, SH-SY5Y cells transfected with PPARδ siRNA had reduced viability in response to ATRA, compared to control SCR siRNA-treated cells (Figure 4B); in the control cells, viability increased in response to ATRA, as is frequently observed (see Figure S1B for example). PPARδ knockdown alone had no significant effect on apoptosis, but had a small effect of increasing apoptosis in combination with ATRA doses of 8 µM or more (P = 0.014; Figure 4C).

Thus, PPARδ inhibition reduced viability and increased cell death after treatment with ATRA, supporting the hypothesis that PPARδ mediates survival in ATRA-treated cells. To test this hypothesis further, we pre-treated SH-SY5Y cells with a PPARδ agonist (0.1 µM L165-041), predicting that it would rescue cells from death mediated by a combination of 5-LO inhibition and ATRA. In viability experiments, the PPARδ agonist did indeed increase the IC_50_ of celecoxib/ATRA and MK886/ATRA combinations, although overall survival at the highest concentrations of ATRA (10–15 µM) was not affected (Table 2).

### Differential PPARδ Isoform Expression Affects the Balance Between Cell Death and Survival

**Figure 5:**
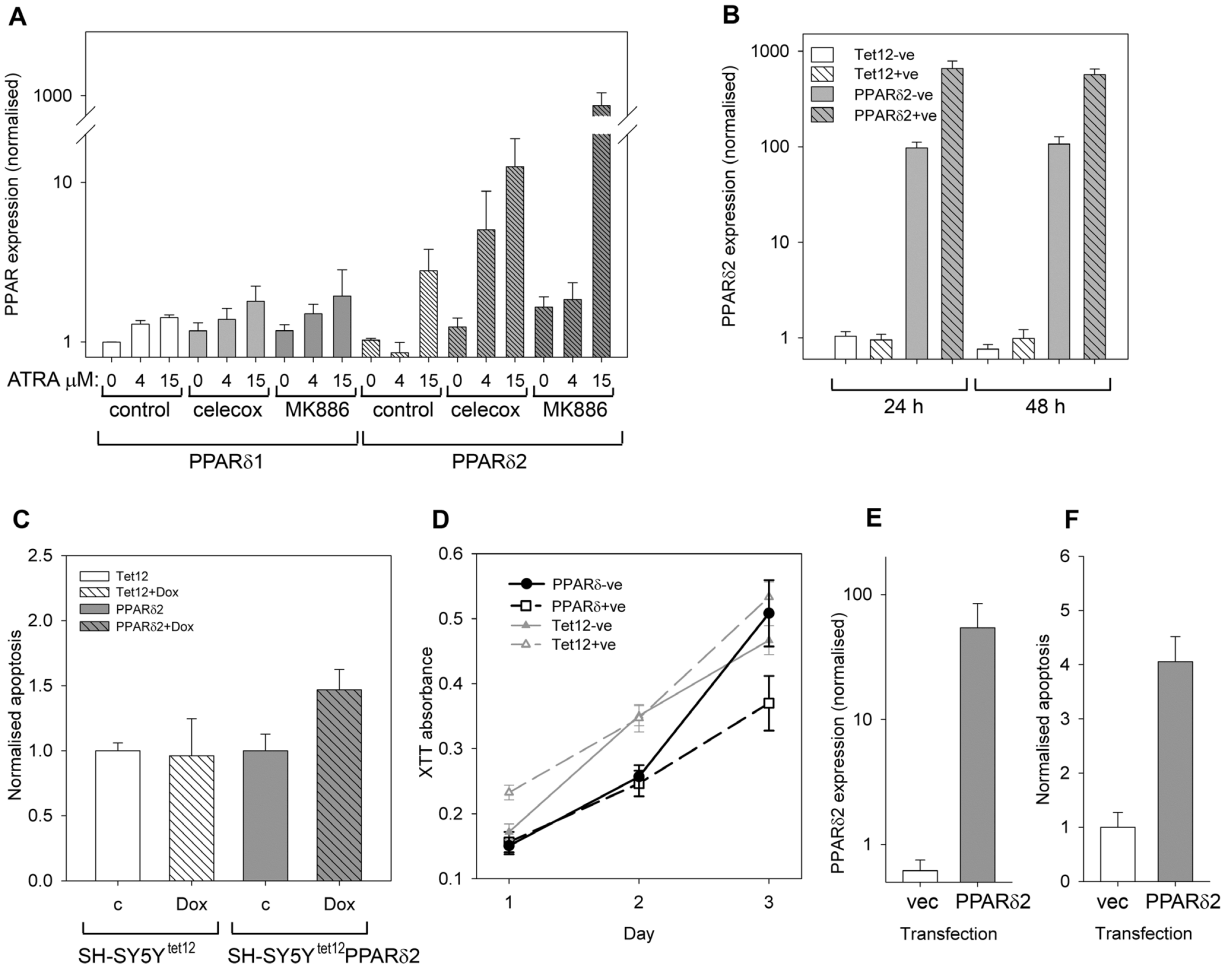
Expression of PPARδ isoforms after 5-LO inhibition and treatment. (**A**) PPARδ1 and PPARδ2 (bars with diagonal lines) mRNA expression after treatment with 0, 4 µM or 15 µM ATRA in the presence of control vehicle (no shading), 22 µM celecoxib (light grey shading) or 1 µM MK886 (dark grey shading). PPARδ1 mRNA expression increased with ATRA treatment (ANOVA, P = 0.03); PPARδ2 mRNA expression increased significantly after treatment with celecoxib with ATRA or MK886 with ATRA (ANOVA, P = 0.033). (**B**) Expression of PPARδ2 transcripts in SH-SY5Y^tet12^PPARδ2 cells treated with doxycycline (referred to as PPARδ2+ve cells; grey bars, diagonal lines) and without doxycycline (referred to as PPARδ2-ve cells; grey bars; no diagonal lines). Parental cells (SH-SY5Y^tet12^ or Tet12 in the legend) are also shown with (+ve, white bars with diagonal lines) and without (−ve; white bars) doxycycline treatment. (**C**) Apoptosis determined using flow cytometry in PPARδ2-ve and PPARδ2+ve cells after 48 h compared to control SH-SY5Y^tet12^ cells with (Dox; Tet12+ve) and without (c; Tet12-ve) doxycycline. Three experiments, 2–3 replicates per experiment; data were normalised to the mean control (without doxycycline) per experiment for each cell line. Up-regulation of PPARδ2 expression with doxycycline significantly increased apoptosis of SH-SY5Y^tet12^PPARδ2 cells (Welch’s t test on all normalised values t_13.4_ = 2.3, P = 0.037), but there was no effect on SH-SY5Y^tet12^ cells (t_7.6_ = −0.14, P>0.8). Overall levels of apoptosis (not normalised) increased from a mean of 22.5% in SY5Y^tet12^PPARδ2 cells without doxycycline to 32.3% in cells after induction of PPARδ2. Despite the leaky expression of PPARδ2 in SH-SY5Y^tet12^PPARδ2 cells without doxycycline, levels of apoptosis were not significantly higher than in the parental SH-SY5Y^tet12^ cells (P>0.4). (**D**) Viability, measured by XTT assays for the PPARδ2-ve (•, solid line) cells (no doxycycline), PPARδ2+ve cells (doxycycline; □, dashed line), Tet12-ve (no doxycycline; ▴, solid grey line) and Tet12+ve cells (doxycycline; Δ, dashed grey line) over 48 h. (**E**) Normalised expression of PPARδ2 transcripts in SH-SY5Y cells after transient transfection with the empty pcDNA4/TO vector (vec; white bars) or pcDNA4/TO PPARδ2 (PPARδ2; grey bars). Cells transfected with pcDNA4/TO PPARδ2 had significantly higher expression of PPARδ2 mRNA 48 h after transfection than the control cells transfected with empty pcDNA4/TO vector (Mann Whitney U, P>0.001). (**F**) Apoptosis, shown normalised to the mean vector control in each of three experiments (3–5 replicates per experiment) in SH-SY5Y cells 72 h after transient transfection with the empty pcDNA4/TO vector (vec; white bars) or pcDNA4/TO PPARδ2 (PPARδ2; grey bars).Mean apoptosis (38.8%) in cells transfected with pcDNA4/TO PPARδ2 was significantly higher than the vector-transfected controls (13.8%; t-test, P<0.05). All error bars are±SEM.

Of the five different PPARδ isoforms described, PPARδ1 and PPARδ2 are the most widely expressed in different tissues; current evidence suggests that PPARδ2 is an inhibitory isoform lacking the ligand binding domain, although a translation product has yet to be identified [18]. We measured the expression of both PPARδ1 and PPARδ2 in neuroblastoma cells (Figure 5A) and found that 2 h after treatment with ATRA, PPARδ1 expression increased significantly with ATRA concentration but there was no significant effect of MK886 or celecoxib on ATRA-induced PPARδ1 levels. In contrast, celecoxib or MK886 increased PPARδ2 expression in combination with ATRA (Figure 5A). To determine the effect of PPARδ2 on cell survival and apoptosis, we created a tetracycline-inducible PPARδ2 system in SH-SY5Y cells (SH-SY5Y^tet12^PPARδ2). In the absence of doxycycline, PPARδ2 expression was higher than in the parental SH-SY5Y^tet12^ cells but was significantly increased 24 and 48 h after treatment with doxycycline (Figure 5B). Up-regulation of PPARδ2 expression in the SH-SY5Y^tet12^PPARδ2 cells significantly increased apoptosis after 48 h (Figure 5C). This was confirmed by cell viability assays over the same time scale (Figure 5D). Doxycycline did not increase apoptosis or slow growth in the SH-SY5Y^tet12^ cells (Figure 5C and D). As an additional test, we transiently transfected the empty vector (pcDNA4/TO) or the pcDNA4/TO PPARδ2 construct into SH-SY5Y cells. Using this approach, PPARδ2 mRNA expression after 48 h was lower than in the stable inducible system (Figure 5E) and there was no baseline expression of PPARδ2 mRNA in the pcDNA4/TO controls. As with the inducible system, cells transfected with the PPARδ2 construct had higher levels of apoptosis (Figure 5F).

### PPARδ Expression and Prognostic Significance in Primary Neuroblastoma Tumours

**Figure 6:**
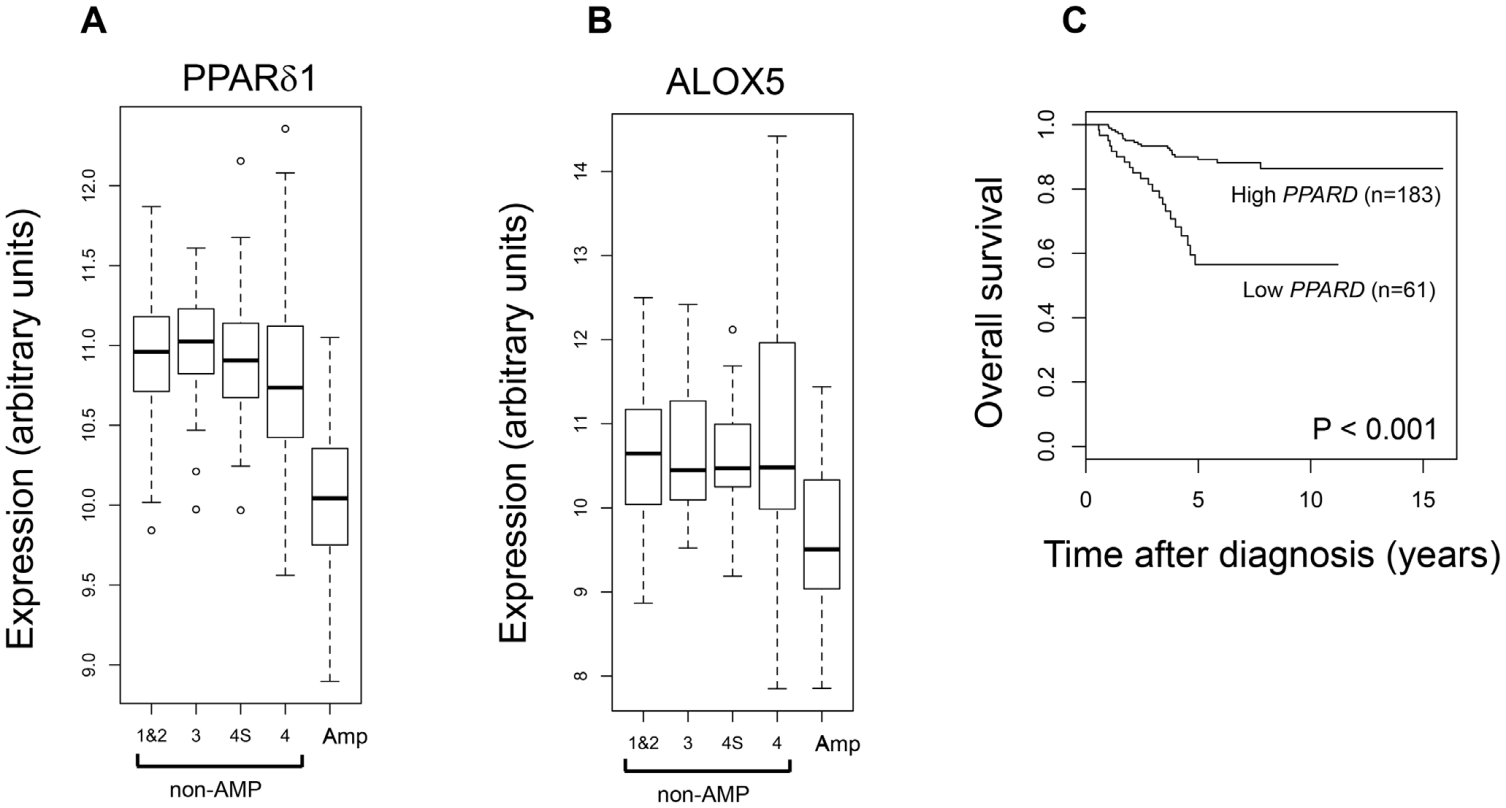
Clinical data from a cohort of 251 primary neuroblastomas. Expression of (**A**) PPARδ1 mRNA and (**B**) ALOX5 (5-LO) mRNA in stages 1–4, 4S and *MYCN* amplified (Amp) groups. (**C**) Kaplan-Meier survival analysis for 251 primary neuroblastomas based on PPARδ1 mRNA expression. Low PPARδ1 or ALOX5 expression was significantly associated with *MYCN* amplification status (Wilcoxon test; P<0.001 for both). For Kaplan-Meier analysis of patient survival, a cut-off at the first quartile was used to distinguish between low PPARδ1 and high PPARδ1 expression: low PPARδ1 expression defined in this way was significantly associated with poor survival (Log Rank test, P<0.001). Multivariate analysis with: age at diagnosis, *MYCN* amplification status, stage and PPARδ1 expression showed that PPARδ1 expression did not contribute significantly (P = 0.866). Box and whisker plots show the median (horizontal line), 25th and 75^th^ percentiles (box horizontal boundaries), with the whiskers above and below extending to the most extreme data point>1.5 times the interquartile range from the box. Outliers are shown as circles.

The *in vitro* PPARδ expression, knockdown and agonist/antagonist studies suggest that PPARδ may have a role in the survival of tumour cells *in vivo*. To address this question, microarray data from a cohort of 251 neuroblastoma primary tumours [33] were analysed. The expression of PPARδ1 and ALOX5 were examined across the following groups: stages 1 & 2, stage 3, stage 4, stage 4S and *MYCN* amplified neuroblastoma. Low PPARδ1 or ALOX5 expression was significantly associated with *MYCN* amplification status (Figure 6A and B), and low PPARδ1 expression was significantly associated with poor survival (Figure 6C). However, when multivariate analysis was carried out, with: age at diagnosis, *MYCN* amplification status, stage and PPARδ1 expression, the independent contribution of PPARδ1 was not significant, suggesting that the prognostic significance of low PPARδ1 was due to its strong correlation with *MYCN* amplification status.

## Discussion

AA has opposing effects on cell survival: inhibition of PLA2 is associated with decreased survival and motility [34], but AA release and its subsequent accumulation after treatment with cytotoxic agents has been suggested to sensitise cells to apoptosis [35]. Our study, alongside others [11], [12], has shown that combining RA with celecoxib synergistically promotes cell death of neuroblastoma cells *in vitro*. It is likely that inhibition of AA metabolism by cyclooxygenases and lipooxygenases promotes cell death by two mechanisms: firstly by preventing the synthesis of mediators of cell survival and, secondly, by promoting an accumulation of AA [36]. Indeed, combinations of inhibitors of AA metabolism (celecoxib) and DNA-damaging drugs that promote AA release, promotes cell death effectively *in vitro* and *in vivo* [10]. It has recently been shown that activated caspase-3 from dying cells promotes AA release via PLA2 in surrounding cells, thus promoting the regrowth of irradiated tumour cells [37], indicating that targeting AA signalling after drug treatment may minimise drug resistance.

**Figure 7:**
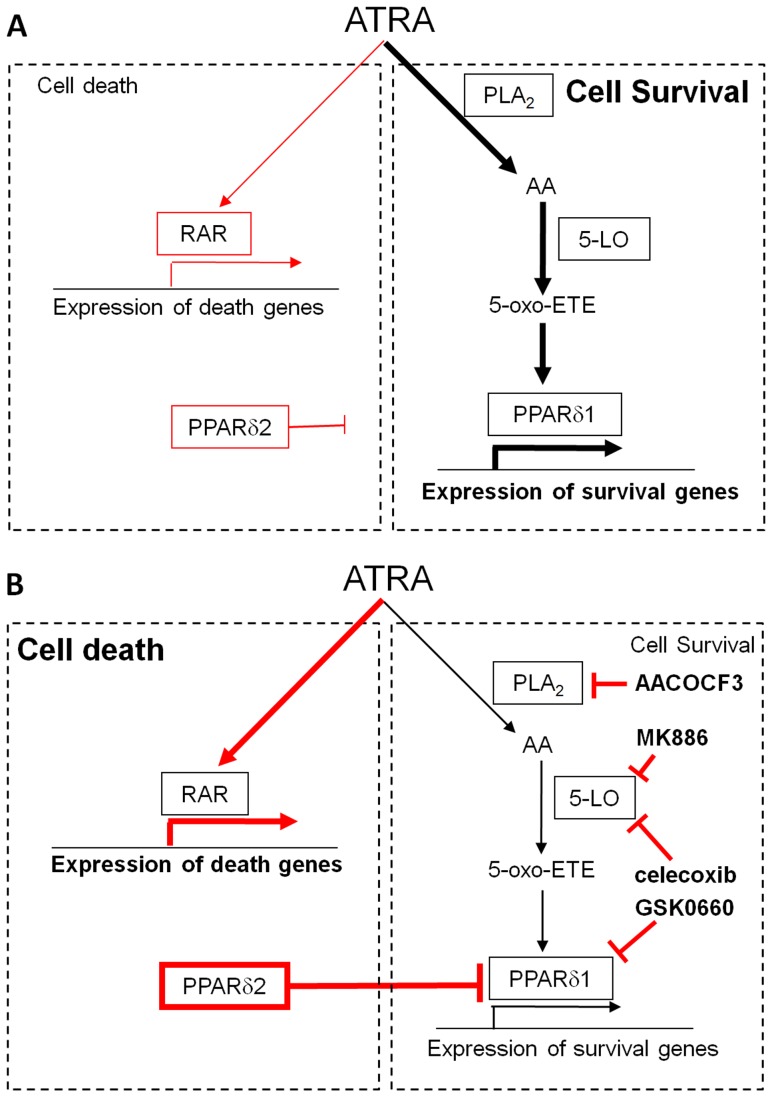
Model of ATRA-stimulated cell-survival signalling in neuroblastoma. (**A**) ATRA cell-survival signalling is proposed to be mediated through PLA2-dependent AA release and metabolism to lipoxygenase products such as 5-oxo-ETE, which activate PPARδ1 to promote expression of survival genes. We suggest that the expression of inhibitory PPARδ2 transcripts is suppressed when this pathway is activated. (**B**) When the PLA2/5-LO/PPARδ pathway is inhibited, we propose that the expression of genes facilitating cell survival is prevented, a process facilitated by up-regulation of PPARδ2 and PPARδ2-mediated suppression, allowing the expression of either stress-induced or RAR-induced (or both) death genes to predominate and drive cell death.

We used small-molecule inhibitors targeted against cyclooxygenases and lipoxygenases in combination with ATRA to elucidate whether AA metabolism promotes cell survival in neuroblastoma cells. Previous studies suggest that PGE2 is an important mediator of RA-induced cell survival signalling [38]–[40]. However, later studies, including this study, have shown that exogenous PGE2 does not affect ATRA and celecoxib-induced cell death [11]. Results from a range of experimental approaches support a COX2-independent mechanism for RA mediated cell survival signalling. The FLAP inhibitor MK886, prevents 5-LO activation and consistently reduced cell viability in combination with RA, suggesting that LO metabolites are important mediators of cell survival after RA treatment. This is also consistent with findings that celecoxib is a potent inhibitor of 5-LO [29] and synergistically promotes cell death in combination with RA. In B cells, 5-oxo-ETE is a major product of AA metabolism which accumulates as a result of oxidative stress. 5-oxo-ETE production is promoted through increased AA production and also increasing levels of NADP+, a cofactor for 5-HEDH [41]. Furthermore, 5-oxo-ETE production is associated with cell survival after treatment with cytotoxic agents in prostate cancer cells [42], [43]. In our study, 5-oxo-ETE partially rescued cells from a combination of RA and celecoxib or MK886 (summarised in Figure 7), suggesting that lipoxygenase metabolites such as 5-oxo-ETE mediate RA-induced cell-survival signalling. Although previous studies have suggested that ATRA is a potent PPARδ ligand [13], subsequent work has not been able to demonstrate binding of ATRA to PPARδ, PPRE-reporter activation by 0.3 µM ATRA or increased ATRA-mediated expression of PPARδ target genes [15]–[17]. In this study we found that ATRA or 5-oxo-ETE treatment activated PPARδ-mediated PPRE-reporter activity in neuroblastoma cells. In contrast to earlier work, the concentrations of ATRA (1 µM–15 µM) we used are high and likely to induce AA release as a result of cell stress resulting from physical effects of ATRA on membranes.

The role of PPARδ in survival responses of ATRA-treated cells is supported by experiments with siRNA-mediated knockdown of PPARδ1 and by the PPARδ antagonist that reduced cell survival in combination with ATRA, effects also observed with celecoxib and MK886. Furthermore, a PPARδ agonist abrogated the effects of celecoxib or 5-LO inhibition.

PPARδ performs a complex role co-ordinating the activity of other PPARs via ligand-independent transcriptional repression at PPREs [44]. Since we found consistent increases in reporter activity after treatment with ATRA or 5-oxo-ETE, which decreased after 5-LO inhibition, this suggests that, at least in part, cell survival signals rely on ligand activation of PPARδ1. Additionally, the putative dominant-negative isoform, PPARδ2, was markedly induced at the mRNA level after celecoxib/ATRA treatment. A dominant-negative role for PPARδ2 was also supported by the finding that ectopic PPARδ2 expression increased apoptosis and reduced proliferation of neuroblastoma cells. In our model, the effects of PPARδ2 were observed after 72 h, and this is not consistent with the levels of cell death observed after 24 h with the drug combinations. However, PPARδ2 up-regulation may function to prolong the suppression of PPARδ1-mediated cell survival and growth signalling. We also speculate that since 5-LO inhibitors induced PPARδ2, PPARδ2 might be repressed in a ligand-dependent manner by PPARδ1.

Understanding the balance of cell-survival and cell-death signalling pathways *in vivo* will help determine the reasons for treatment failure and optimise therapeutic strategies with RA in neuroblastoma patients. Low PPARδ and ALOX5 expression was associated with *MYCN* amplification in a cohort of 251 primary neuroblastoma tumours. Although low PPARδ1 expression was significantly associated with poor prognosis, it was not an independent prognostic indicator due the correlation with *MYCN* amplification. It is possible that PPARδ1 expression is up-regulated in tumour cells in response to patient treatment and this remains to be investigated. It should also be noted that MYCN expression is down regulated in neuroblastoma cells after treatment with RA [2], so if PPARδ1 expression is repressed directly or indirectly by MYCN, its potential up-regulation in response to RA would act as a compensatory cell-survival and cell-growth mechanism.

The mechanism linking low PPARδ1 and ALOX5 to *MYCN* amplification status in neuroblastoma needs to be investigated, and the study raises questions over the potential role of PPARδ1 in promoting cell survival signalling in neuroblastoma (Figure 7). Clearly, if these ideas are supported by future work, this will lead to new targets for increasing the efficiency of RA, particularly PPARδ and the enzymes involved AA metabolism. Celecoxib also promotes death of neuroblastoma cells synergistically in combination with cytotoxic drugs *in vitro* and *in vivo* [10], therefore it is important to determine if PPARδ signalling is a common mechanism of drug resistance in cancer.

In summary, the results of this study in neuroblastoma cells suggest that high levels of ATRA induce a survival response mediated by the metabolism of AA by lipoxygenases, particularly 5-LO. The experimental data implicate the 5-LO product 5-oxo-ETE as a ligand activating PPARδ1 to promote survival. Blocking this signalling pathway at the level of AA release, 5-LO-mediated metabolism or PPARδ1 antagonism promoted cell death. Furthermore, ligand-dependent and ligand-independent- negative feedback relationships between PPARδ1 and a dominant-negative PPARδ isoform, PPARδ2 may have a role in the control of survival signalling. It will be important to test these ideas using other cytotoxic drugs which promote AA release and in other cell types. Finally, the possibility of a more-general role in cell survival for PPARδ1 with negative-feedback regulation by a dominant-negative isoform needs further investigation.

## Supporting Information

Figure S1 **The role of COX2 in retinoic acid mediated cell death.**
(TIF)

Figure S2 **PPARδ expression and PPRE activation in SH-SY5Y cells.**
(TIF)

Figure S3 **Western blot for cleaved caspase-3.**
(TIF)

Figure S4 **Western blot for COX2.**
(TIF)

Table S1 **Viability of NB69 and NGP neuroblastoma cells in response to ATRA in combination with celecoxib, or inhibitors of PLA2 and 5-LO.**
(DOCX)

Table S2 **Viability of NB69, NGP and SH-SY5Y neuroblastoma cells in response to ATRA in combination with the dual COX1 and COX2 inhibitor diclofenac, or inhibitors of 12-LO and 15-LO.**
(DOCX)

Text S1 **The role of COX2 in cell survival.**
(DOCX)

Text S2 **12-LO and 15-LO inhibitors in combination with ATRA.**
(DOCX)
