# Supplementary figures and images for: Cell Survival Signalling through PPARδ and Arachidonic Acid Metabolites in Neuroblastoma
Source: PLoS One. 2013 Jul 9;8(7):e68859. doi: 10.1371/journal.pone.0068859 (PMC3706415; doi:10.1371/journal.pone.0068859)

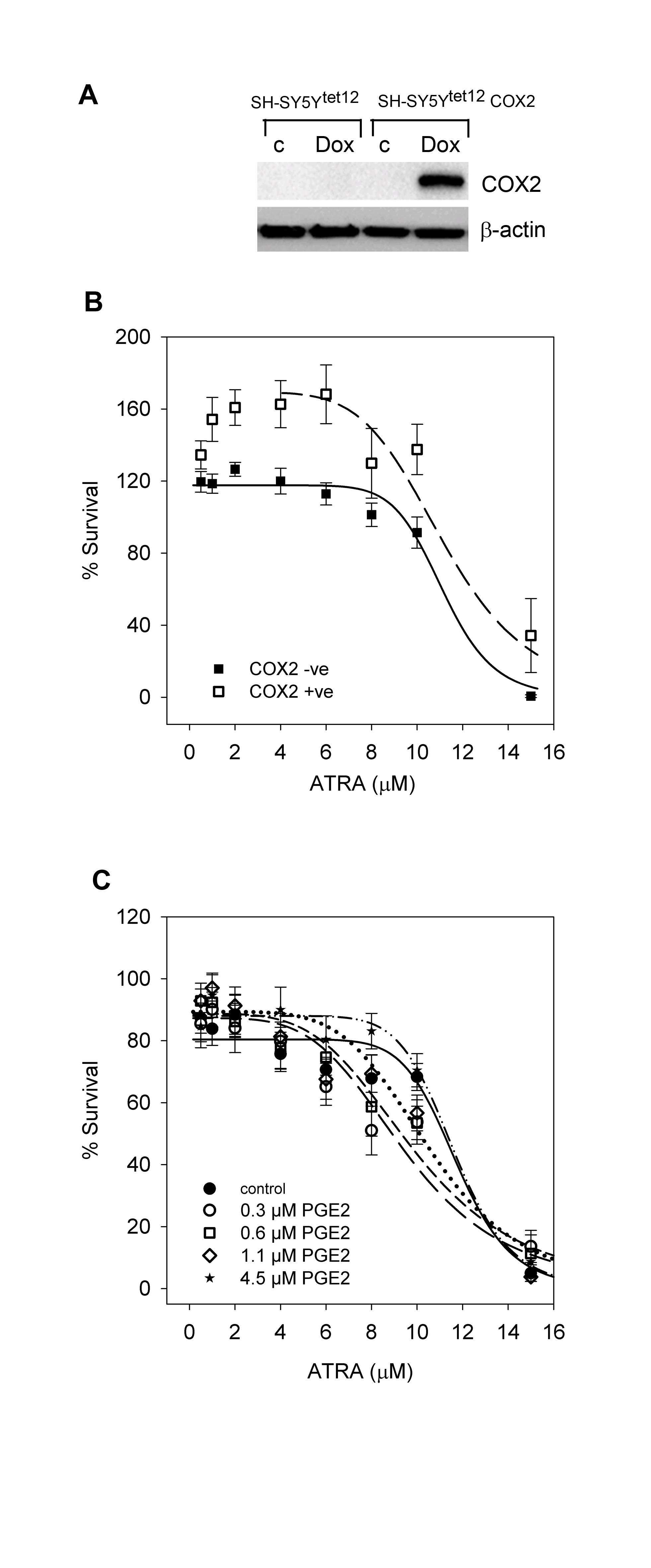

Supplement: Figure S1 — The role of COX2 in retinoic acid mediated cell death. (TIF) [file pone.0068859.s001.tif]

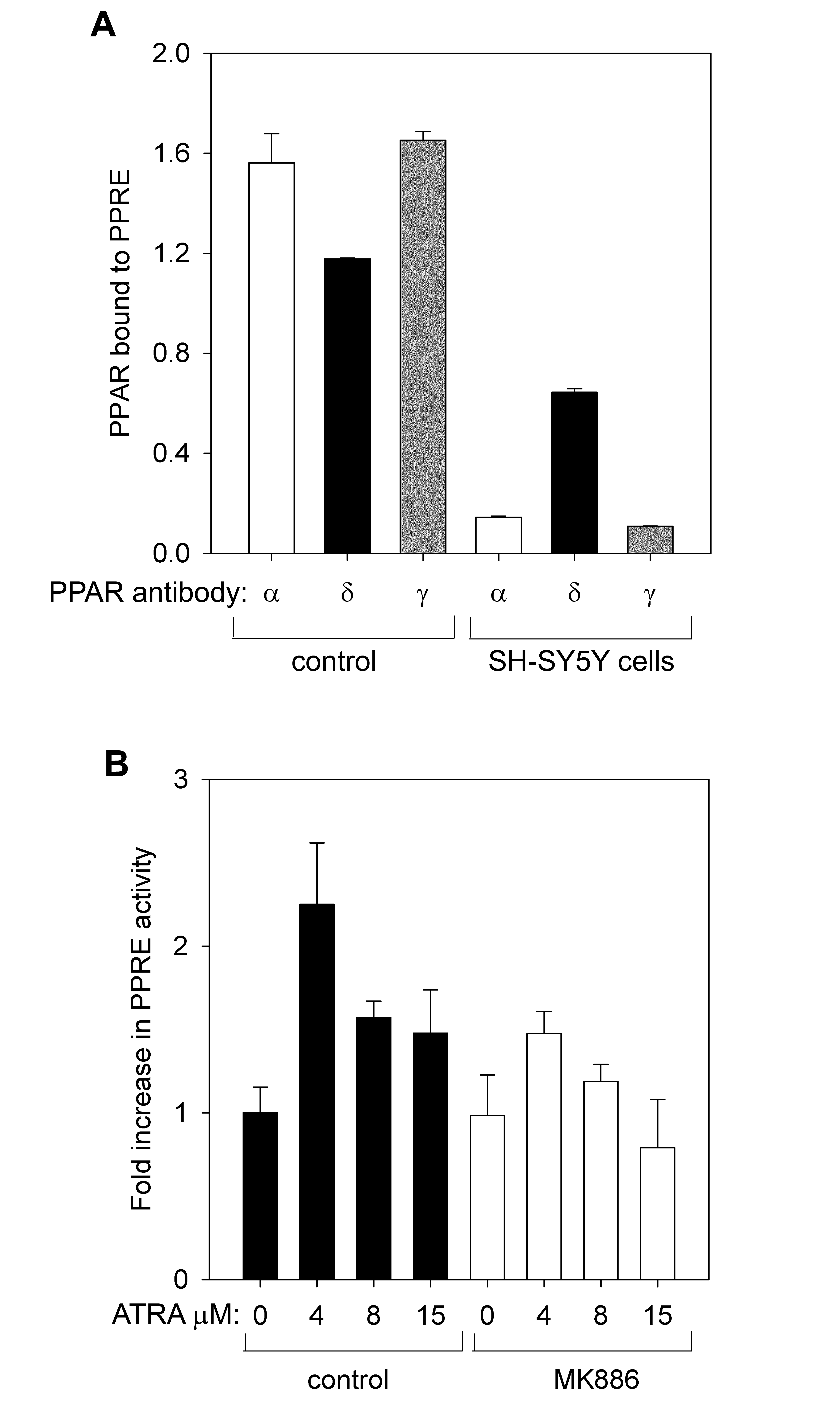

Supplement: Figure S2 — PPARδ expression and PPRE activation in SH-SY5Y cells. (TIF) [file pone.0068859.s002.tif]

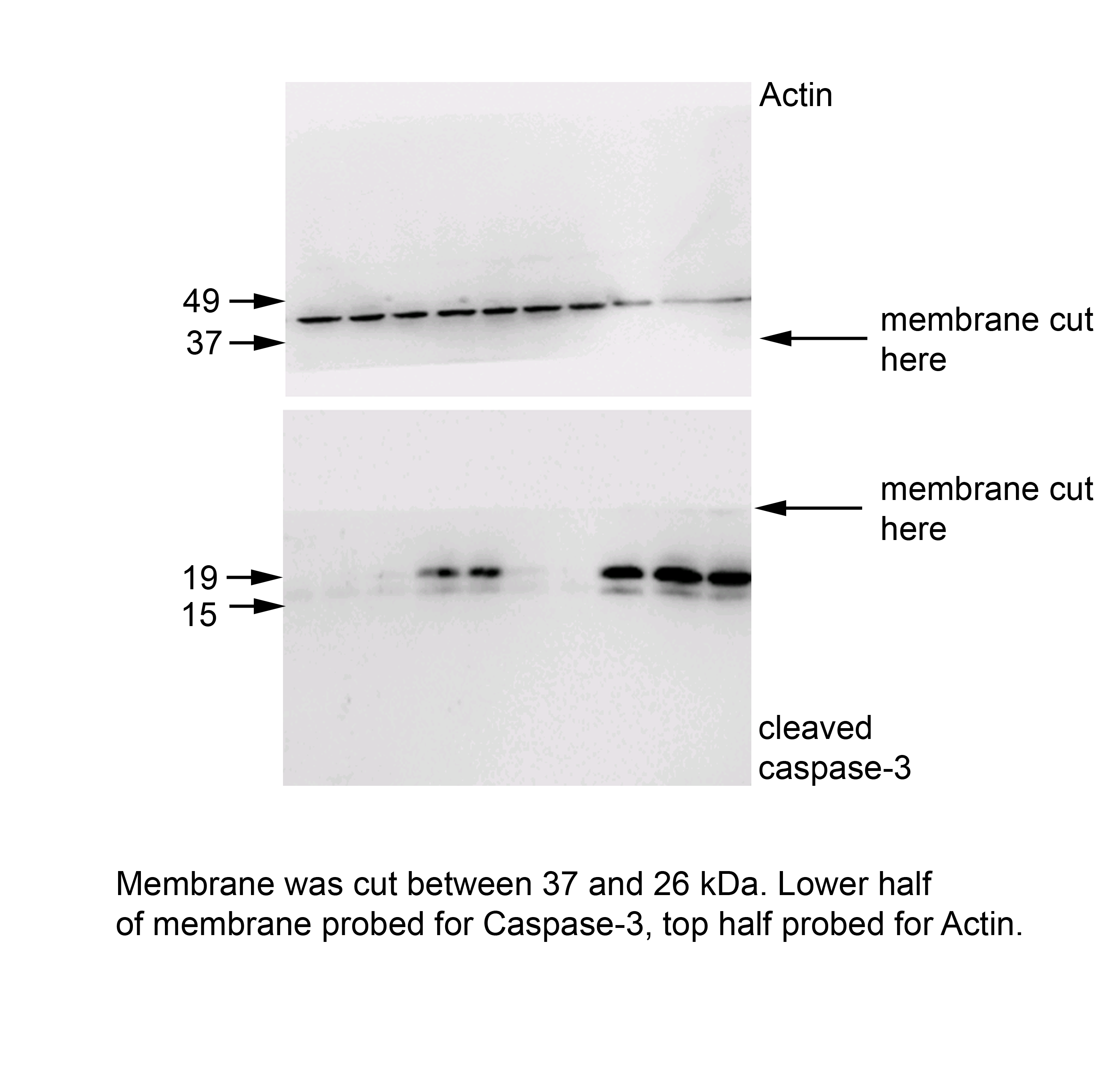

Supplement: Figure S3 — Western blot for cleaved caspase-3. (TIF) [file pone.0068859.s003.tif]

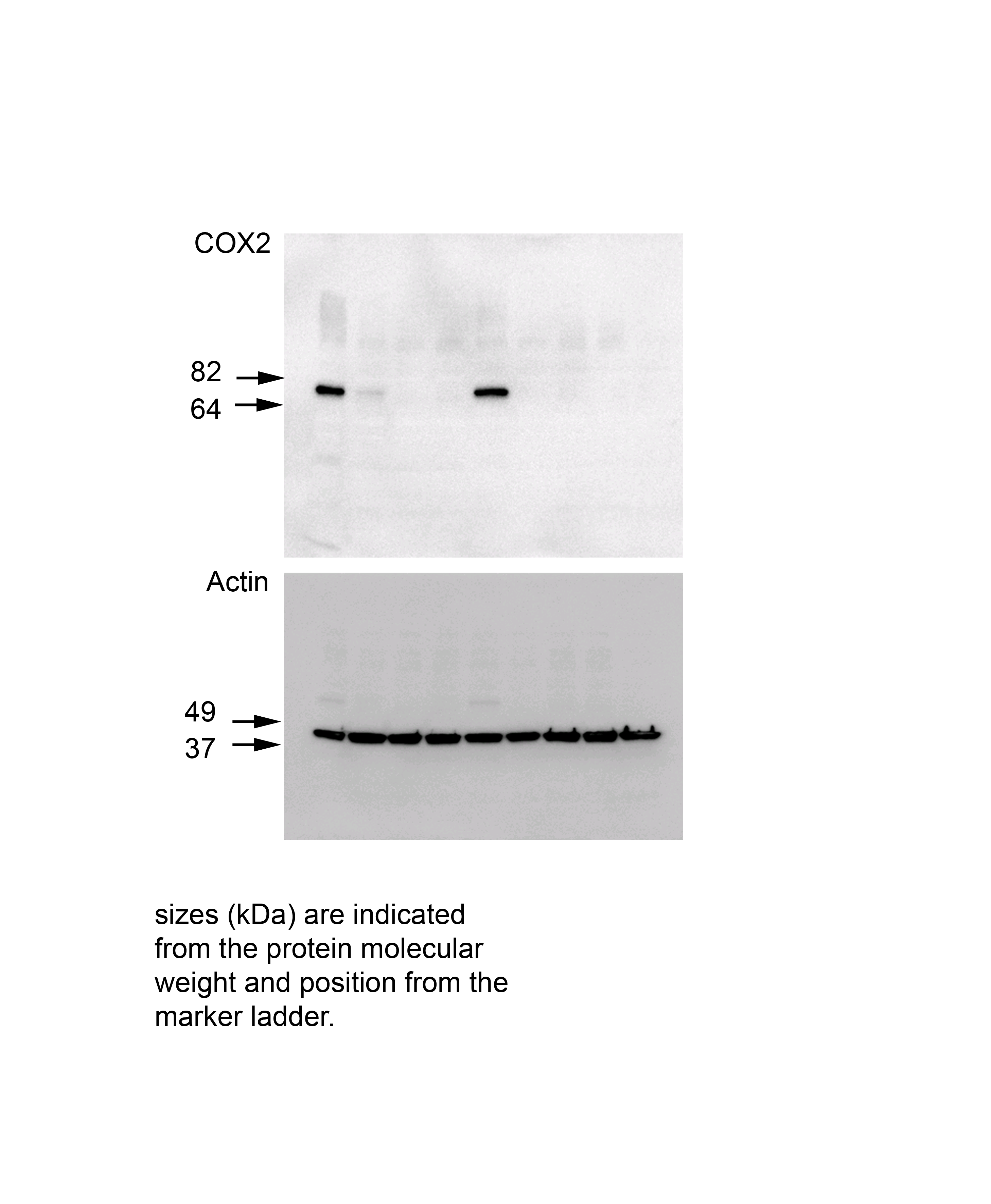

Supplement: Figure S4 — Western blot for COX2. (TIF) [file pone.0068859.s004.tif]
