# Supplementary material for: Cell Survival Signalling through PPARδ and Arachidonic Acid Metabolites in Neuroblastoma
Source: PLoS One. 2013 Jul 9;8(7):e68859. doi: 10.1371/journal.pone.0068859 (PMC3706415; doi:10.1371/journal.pone.0068859)
Supplement: Table S1 — Viability of NB69 and NGP neuroblastoma cells in response to ATRA in combination with celecoxib, or inhibitors of PLA2 and 5-LO. (DOCX) [file pone.0068859.s005.docx]

**Table S1**

**Table S1: Viability of NB69 and NGP neuroblastoma cells in response to ATRA in combination with celecoxib, or inhibitors of PLA2 and 5-LO.** Treatment combinations were tested on NB69 and NGP neuroblastoma cells. Cells were pre-treated with celecoxib or inhibitor at concentrations specified in the Table prior to treatment with a range of ATRA doses. Cell viabilities (% of control) for cells (means ± SEM) treated with the inhibitor alone are given alongside the IC_50_ of ATRA in combination with the inhibitor. The IC_50_ was calculated from data where 0% viability was observed at maximal ATRA dose (15 µM). For celecoxib and ATRA viability data, the combination indices (CI) were calculated: for NB69 cells 22µM celecoxib was synergistic at 10 and 15 µM ATRA (CI= 0.789 and 0.072 respectively). For NGP cells synergism was observed at 6, 8, 10 and 15 µM ATRA (CI=0.125, 0.316, 0.020 and 0.132, respectively).

| **Drug/ concentration** | **Target** | **Cell line** | **% Viability drug alone** | **IC_50_ (µM) ATRA + inhibitor** |
| --- | --- | --- | --- | --- |
| ATRA |  | NB69 | 85.51 ± 18.22%* |  |
|  |  | NGP | 89.25 ± 10.26%* |  |
| Celecoxib  22µM | COX2/ 5-LO | NB69 | 93.13 ± 9.85 | 4.36 ± 12.27 |
|  |  | NGP | 71.13 ± 17.88 | 2.31 ± 3.23 |
| AACOCF3  10µM | PLA_2_ | NB69 | 29.83 ± 11.15 | 4.00 ± 1.33 |
|  |  | NGP | 51.35 ± 23.37 | 2.87 ± 1.03 |
| MK886  1µM | FLAP (5-LO) | NB69 | 51.7 ± 11.6 | 1.33 ± 3.67 |
|  |  | NGP | 150.1± 14.5 | 4.86 ± 2.02 |

*Viability at maximal ATRA (15 µM)
