# Supplementary material for: Cell Survival Signalling through PPARδ and Arachidonic Acid Metabolites in Neuroblastoma
Source: PLoS One. 2013 Jul 9;8(7):e68859. doi: 10.1371/journal.pone.0068859 (PMC3706415; doi:10.1371/journal.pone.0068859)
Supplement: Table S2 — Viability of NB69, NGP and SH-SY5Y neuroblastoma cells in response to ATRA in combination with the dual COX1 and COX2 inhibitor diclofenac, or inhibitors of 12-LO and 15-LO. (DOCX) [file pone.0068859.s006.docx]

**Table S2**

**Table S2: Viability of NB69, NGP and SH-SY5Y neuroblastoma cells in response to ATRA in combination with the dual COX1 and COX2 inhibitor diclofenac, or inhibitors of 12-LO and 15-LO.** Cells were pre-treated with inhibitor at concentrations specified in the Table prior to treatment with a range of ATRA doses. Cell viabilities (% of control) for cells (means ± SEM) treated with the inhibitor alone are given alongside the IC_50_ of ATRA in combination with the inhibitor. Where % viability is not given in the ATRA+inhibitor column, the IC_50_ was calculated from data where 0% viability was observed at maximal ATRA dose (15 µM). NC = not calculated: curve fitting procedures could not give an accurate IC_50_ when there was little effect of the combination on cell viability.

| **Drug/**  **concentration** | **Target** | **Cell line** | **% Viability drug alone** | **IC_50_ (μM) ATRA + inhibitor** |
| --- | --- | --- | --- | --- |
| Diclofenac  400 µM | COX1/ COX2 | NB69 | 30.24 ± 7.71 | NC  30.64 ± 5.37%* |
|  |  | NGP | 42.68 ± 12.00 | 7.27 ± 5.06  21.59 ± 4.37%* |
|  |  | SH-SY5Y | 47.5% ± 14.3 | 9.8 ± 2.1  28.0% ± 5.0* |
| Baicalein  1 µM | 12-LO | NB69 | 102.75 ± 10.17 | NC  69.63 ± 5.45%* |
|  |  | NGP | 87.93 ± 15.38 | NC  69.82 ± 8.57%* |
|  |  | SH-SY5Y | 142.1 ± 7.7 | 8.96 ± 6.01  60.26% ± 6.64* |
| PD-146176  0.3 µM | 15-LO | NB69 | 73.2 ± 17.4 | 5.63 ± 1.84 |
|  |  | NGP | 33.1 ± 5.4 | 9.86 ± 0.67 |
|  |  | SH-SY5Y | 47.2 ± 11.4 | 9.92 ± 0.27 |

*Viability at maximal ATRA (15 µM)
