# Supplementary material for: Cell Survival Signalling through PPARδ and Arachidonic Acid Metabolites in Neuroblastoma
Source: PLoS One. 2013 Jul 9;8(7):e68859. doi: 10.1371/journal.pone.0068859 (PMC3706415; doi:10.1371/journal.pone.0068859)
Supplement: Text S1 — The role of COX2 in cell survival. (DOCX) [file pone.0068859.s007.docx]

**Text S1: The role of COX2 in cell survival**

To investigate the role of AA metabolism by COX2 in promoting cell survival after ATRA treatment, we used three approaches: treatment of cells with the dual COX1 and COX2 inhibitor diclofenac, tetracycline-inducible over-expression of COX2 in SH-SY5Y neuroblastoma cells, and supplementation of growth medium with prostaglandin E2 (PGE2), a downstream metabolite of the COX2 product prostaglandin H2 (PGH2).

**COX2 inhibition with diclofenac**

In contrast to celecoxib, COX inhibition using diclofenac did not synergistically promote cell death (viability data of Table S2). For SH-SY5Y cells, the IC_50_ of ATRA in combination with diclofenac was 9.8 ± 2.1 µM. Treatment with 400 µM diclofenac alone decreased viability to 47.5% ± 14.3 and in combination with ATRA there was only a marginal effect on viability, decreasing cell survival only to 28.0% ± 5.0 at the highest concentration of ATRA (15µM; Table S2). Similar results were obtained with NGP and NB89 neuroblastoma cells (Table S2).

**Over-expression of COX2 in SH-SY5Y cells**

We introduced a tetracycline COX2 inducible system into the SH-SY5Y^tet12^ cell line [1], generating SH-SY5Y^tet12^COX2 cells in which COX2 was inducible with tetracycline antibiotics. In this model, COX2 protein was increased by 40 to 170-fold after treatment with 1 μg/ml doxycycline (Figure S1A). We refer to SH-SY5Y^tet12^COX2 cells grown with doxycycline as COX2+ve and without doxycycline as COX2-ve. The COX2 activity of the cells was validated by measuring PGE2 levels in the culture medium of the cells using an enzyme-linked immunosorbent assay kit (Cayman Chemicals, Ann Arbour, MI, USA); PGE2 levels were > 20-fold higher in the COX2+ve cells compared to the COX2 –ve cells (ANOVA; P<0.001). Increased COX2 expression in SH-SY5Y^tet12^COX2 cells increased cell survival at low levels of ATRA and celecoxib but, after taking this marked hormetic effect into account, had no apparent effect on the dose-response kinetics of sensitivity to ATRA and celecoxib (Figure S1B).

**The role of PGE2 in cell survival**

To assess a possible role of PGE2 in cell survival, 0.3 - 4.5 µM PGE2 was added to SH-SY5Y cells prior to treatment with 22 μM celecoxib and ATRA. If celecoxib-mediated reduction in PGE2, resulting from COX2 inhibition, was responsible for cell death from celecoxib in combination with ATRA, we would predict cells to be rescued by the addition of exogenous PGE2 to the culture medium. However, low concentrations (0.3 - 1.1 µM) reduced the IC_50_ of celecoxib with ATRA compared to the control (Figure S1C) whereas high concentrations had little effect (Figure S1C).

Taken together, these results suggest that COX2 is not involved in ATRA-mediated survival signalling and that celecoxib may inhibit alternative enzymes in the AA pathway.

**Reference**

1. Lovat PE, Oliverio S, Ranalli M, Corazzari M, Rodolfo C, et al. (2002) GADD153 and 12-lipoxygenase mediate fenretinide-induced apoptosis of neuroblastoma. Cancer Res 62: 5158-5167.
