# Supplementary material for: Cell Survival Signalling through PPARδ and Arachidonic Acid Metabolites in Neuroblastoma
Source: PLoS One. 2013 Jul 9;8(7):e68859. doi: 10.1371/journal.pone.0068859 (PMC3706415; doi:10.1371/journal.pone.0068859)
Supplement: Text S2 — 12-LO and 15-LO inhibitors in combination with ATRA. (DOCX) [file pone.0068859.s008.docx]

**Text S2: 12-LO and 15-LO inhibitors in combination with ATRA**

To investigate a role of 12-LO and 15-LO in cell survival signalling with retinoic acid, cells were treated with sub-lethal doses of the 12-LO inhibitor baicalein (1 µM) or the 15-LO inhibitor PD-146176 (0.3 µM) [[1](#_ENREF_1)]. Compared to MK886, 12-LO inhibition in combination with ATRA was relatively ineffective at reducing viability in any of the three cell lines tested (Table S2). Although all three cell lines were sensitised to ATRA-induced cell death by the 15-LO inhibitor, IC50 values for the effects of ATRA in combination with PD-146176 were greater than those observed for combinations of MK886 and ATRA, and this inhibitor on its own produced higher levels of cell death than the other inhibitors used. Overall, in combination with ATRA, the 15-LO inhibitor was not as effective as the 5-LO inhibitor at the doses used, with the IC50 of ATRA higher in the presence of PD146176 compared to MK886; levels of apoptosis were also higher in MK886 treated cells (Table 1 and Table S2). In contrast to 5-LO and 12-LO, the expression of 15-LO protein is undetectable by Western blotting in SH-SY5Y cells [[1](#_ENREF_2)] and we interpret these results to suggest that 5-LO activity may be more important than 12-LO and 15-LO in promoting cell survival after ATRA treatment in neuroblastoma.

**Reference**

1. Lovat PE, Ranalli M, Corazzari M, Raffaghello L, Pearson AD, et al. (2003) Mechanisms of free-radical induction in relation to fenretinide-induced apoptosis of neuroblastoma. J Cell Biochem 89: 698-708.
